# Identification of programmed cell death-related gene signature and associated regulatory axis in cerebral ischemia/reperfusion injury

**DOI:** 10.3389/fgene.2022.934154

**Published:** 2022-08-04

**Authors:** Jun Shu, Lu Yang, Wenshi Wei, Li Zhang

**Affiliations:** Department of Neurology, Cognitive Disorders Center, Huadong Hospital Affiliated to Fudan University, Shanghai, China

**Keywords:** apoptosis, pyroptosis, necroptosis, cerebral ischemia/reperfusion (I/R) injury, competing endogenous RNA (ceRNA) network

## Abstract

**Background:** Numerous studies have suggested that programmed cell death (PCD) pathways play vital roles in cerebral ischemia/reperfusion (I/R) injury. However, the specific mechanisms underlying cell death during cerebral I/R injury have yet to be completely clarified. There is thus a need to identify the PCD-related gene signatures and the associated regulatory axes in cerebral I/R injury, which should provide novel therapeutic targets against cerebral I/R injury.

**Methods:** We analyzed transcriptome signatures of brain tissue samples from mice subjected to middle cerebral artery occlusion/reperfusion (MCAO/R) and matched controls, and identified differentially expressed genes related to the three types of PCD(apoptosis, pyroptosis, and necroptosis). We next performed functional enrichment analysis and constructed PCD-related competing endogenous RNA (ceRNA) regulatory networks. We also conducted hub gene analysis to identify hub nodes and key regulatory axes.

**Results:** Fifteen PCD-related genes were identified. Functional enrichment analysis showed that they were particularly associated with corresponding PCD-related biological processes, inflammatory response, and reactive oxygen species metabolic processes. The apoptosis-related ceRNA regulatory network was constructed, which included 24 long noncoding RNAs (lncRNAs), 41 microRNAs (miRNAs), and 4 messenger RNAs (mRNAs); the necroptosis-related ceRNA regulatory network included 16 lncRNAs, 20 miRNAs, and 6 mRNAs; and the pyroptosis-related ceRNA regulatory network included 15 lncRNAs, 18 miRNAs, and 6 mRNAs. Hub gene analysis identified hub nodes in each PCD-related ceRNA regulatory network and seven key regulatory axes in total, namely, lncRNA Malat1/miR-181a-5p/Mapt, lncRNA Malat1/miR-181b-5p/Mapt, lncRNA Neat1/miR-181a-5p/Mapt, and lncRNA Neat1/miR-181b-5p/Mapt for the apoptosis-related ceRNA regulatory network; lncRNA Neat1/miR-181a-5p/Tnf for the necroptosis-related ceRNA regulatory network; lncRNA Malat1/miR-181c-5p/Tnf for the pyroptosis-related ceRNA regulatory network; and lncRNAMalat1/miR-181a-5p for both necroptosis-related and pyroptosis-related ceRNA regulatory networks.

**Conclusion:** The results of this study supported the hypothesis that these PCD pathways (apoptosis, necroptosis, pyroptosis, and PANoptosis) and crosstalk among them might be involved in ischemic stroke and that the key nodes and regulatory axes identified in this study might play vital roles in regulating the above processes. This may offer new insights into the potential mechanisms underlying cell death during cerebral I/R injury and provide new therapeutic targets for neuroprotection.

## 1 Introduction

Ischemic stroke is one of the leading causes of long-term severe disability and death worldwide, which is usually caused by a permanent or transient local reduction in blood supply to the brain (Campbell and Khatri, 2020; Mendelson and Prabhakaran, 2021). Currently, the most effective strategy for ischemic stroke patients is to restore cerebral blood flow in a timely manner through drugs and surgery (Herpich and Rincon, 2020). However, injury to brain tissue caused by ischemia and hypoxia is further aggravated following the short-term recovery of blood perfusion, which is known as cerebral ischemia/reperfusion (I/R) injury. The mechanism by which cerebral ischemia/reperfusion injury occurs has not been fully elucidated. Nonetheless, a growing body of evidence suggests that the overproduction of ROS and activation of inflammation and immune responses might be involved, which ultimately trigger cell death, including apoptosis, necroptosis, and pyroptosis (Eltzschig and Eckle, 2011; Jurcau and Simion, 2021). There is thus a need for a comprehensive understanding of the mechanisms underlying cell death during cerebral ischemia/reperfusion (I/R) injury to rescue injured cells, especially injured neurons in the brain, and seek new neuroprotective therapies.

Multiple cell death pathways are currently believed to be involved in cell death in ischemic stroke, among which apoptosis, pyroptosis, and necroptosis are three key programmed cell death (PCD) pathways (Tuo et al., 2022). Apoptosis can be triggered through the intrinsic and/or extrinsic pathway and may contribute to a significant proportion of neuron death following cerebral ischemia/reperfusion (Radak et al., 2017; Datta et al., 2020). Meanwhile, necroptosis is a newly discovered mechanism of cell death that is mainly regulated by receptor-interacting protein kinase 1 (RIPK1), receptor-interacting protein kinase 3 (RIPK3), and mixed-lineage kinase domain-like pseudokinase (MLKL) (Liao et al., 2020). Increasing studies have suggested that necroptosis participates in the pathogenesis of various diseases including ischemia stroke. Studies have also indicated that the inhibition of necroptosis can exert neuroprotective effects after cerebral I/R in mice by reducing cerebral infarct volume and improving motor and cognitive function (Deng et al., 2019; Yao et al., 2021). Pyroptosis is a kind of inflammatory programmed cell death that is characterized by rapid plasma-membrane rupture and the release of proinflammatory intracellular contents as well as cytokines (Yu et al., 2021). Pyroptosis was reported to be triggered by certain inflammasomes and activating caspases and executed by gasdermin family members (Dong et al., 2018). Accumulating evidence has shown that these three PCD pathways participate in the pathogenesis of ischemic stroke and that their inhibition could attenuate ischemic brain injury (Tuo et al., 2022). Recently, further evidence has also shown significant crosstalk among the three PCD pathways (Banoth et al., 2020; Zheng et al., 2020; Karki et al., 2021). Against this background, the concept of PANoptosis was proposed, which is defined as a proinflammatory PCD pathway with key features of pyroptosis, apoptosis, and/or necroptosis that cannot be accounted for by any of these PCD pathways alone (Malireddi et al., 2020; Wang and Kanneganti, 2021). PANoptosis is regulated by the cytoplasmic multimeric protein complex called the PANoptosome, which can participate in the three PCD pathways in parallel (Samir et al., 2020). PANoptosis has been implicated in various conditions, including infection, sterile inflammation, and cancer (Karki et al., 2020; Zheng et al., 2020; Place et al., 2021). A recent study that collected, integrated, and analyzed reports on research on cerebral I/R indicated that PANoptosis is observed in ischemic brain injury (Yan et al., 2022). Despite efforts to reveal the role of PCD pathways in cerebral I/R injury, the mechanisms underlying the involvement of the three PCD pathways in cerebral I/R injury are extremely complicated and remain largely unknown.

In this study, we collected PCD (apoptosis, pyroptosis, and necroptosis)-related genes based on previous literature and related databases, and analyzed transcriptome signatures of brain tissue samples from mice subjected to middle cerebral artery occlusion/reperfusion (MCAO/R) and matched controls to identify differentially expressed genes related to the three types of PCD. We then performed functional enrichment analysis of these differentially expressed PCD-related genes and their potential regulatory axes to explore their potential biological functions and regulatory mechanisms. This bioinformatic analysis might provide new insights into the potential mechanisms underlying cell death during cerebral I/R injury and new therapeutic targets for neuroprotection.

## 2 Materials and methods

### 2.1 Collection of datasets and programmed cell death-related genes

We searched the Gene Expression Omnibus (GEO) database (Barrett et al., 2013) (https://www.ncbi.nlm.nih.gov/geo) using the following terms: “cerebral ischemia–reperfusion OR cerebral ischemia OR ischemia stroke” AND “*Mus musculus*.” We included the gene expression profiling of adult mouse brain tissues after transient focal ischemia at 24 h of reperfusion and matched control samples. Then two datasets, GSE131193 and GSE58720, were downloaded for analysis. The dataset GSE131193 based on the GPL19057 platform is an mRNA high-throughput sequencing series that includes data on contralateral and ipsilateral brain tissues from mice subjected to transient middle cerebral artery occlusion (tMCAO) at different reperfusion timepoints (24 h and 7 days) and matched sham-operated mice. We selected a subset comprising three ipsilateral brain tissues after transient focal ischemia at 24 h of reperfusion and three matched sham-operated mice for analysis. The dataset GSE58720 based on the GPL10787 platform contains microarray gene expression data of brain tissue samples from three MCAO-operated mice at 24 h of reperfusion and three matched sham-operated mice.

For apoptosis-related genes (ARGs), 101 ARGs were downloaded from Reactome Pathway Database (https://reactome.org/) (Jassal et al., 2020) and two were extracted from the literature, thus 103 ARGs were collected (Supplementary Table S1); for necroptosis-related genes (NRGs), twenty-seven NRGs were downloaded from Reactome Pathway Database, eighty-two NRGs were extracted from the literature, after removing the overlapping genes, ninety-three NRGs were obtained (Supplementary Table S2); for pyroptosis-related genes (PRGs), twenty PRGs were downloaded from Reactome Pathway Database, sixty-seven PRGs were extracted from the literature, after removing the overlapping genes, seventy-eight PRGs were obtained for further study (Supplementary Table S3).

### 2.2 Screening strategy for differentially expressed programmed cell death-related genes

Differentially expressed genes (DEGs) of the microarray dataset GSE58720 were identified with NCBI’s GEO2R tool (https://www.ncbi.nlm.nih.gov/geo/geo2r/) using the Limma package. For the sequencing dataset GSE131193, the processed count matrix data was downloaded and differential analysis between tMCAO-operated mice and their matched control was conducted using the “lmFit” and “eBayes” functions in the Limma package (Ritchie et al., 2015). A *p*-value < 0.05 and |log2 fold change (FC)| > 1 were regarded as cut-off criteria for significant DEGs. The common DEGs in common between the GSE58720 dataset and the GSE131193 dataset were intersected with PCD (apoptosis, necroptosis, and pyroptosis)-related genes, respectively, to obtain apoptosis-related DEGs (ARDEGs), necroptosis-related DEGs (NRDEGs), and pyroptosis-related DEGs (PRDEGs). The above results were visualized using the online tool Jvenn (http://jvenn.toulouse.inra.fr/app/index.html) (Bardou et al., 2014).

### 2.3 Functional enrichment analysis

To obtain a better understanding of the biological mechanisms of the differentially expressed PCD-related genes, functional enrichment analysis, including Gene Ontology (GO) and pathway enrichment analysis, were performed using Metascape (http://metascape.org) (Zhou et al., 2019). The Kyoto Encyclopedia of Genes and Genomes (KEGG) (Kanehisa et al., 2017), Reactome (Jassal et al., 2020), and WikiPathways (Martens et al., 2021) databases were used for pathway annotations.

### 2.4 Construction of PCD-related ceRNA networks

To explore the potential regulatory mechanisms of these differentially expressed PCD-related genes, we constructed intricate competing endogenous RNA (ceRNA) networks. First, target microRNAs (miRNAs) of the obtained differentially expressed PCD-related genes were predicted by four independent online databases: TargetScan (Agarwal et al., 2015) (v7.2, http://www.targetscan.org/vert_72/), miRTarBase (Huang et al., 2020) (v8.0, http://mirtarbase.mbc.nctu.edu.tw/php/index.php), StarBase (Li et al., 2014) (http://starbase.sysu.edu.cn/), and miRWalk (Sticht et al., 2018) (http://mirwalk.umm.uni-heidelberg.de/). Only the miRNAs that were shared by any three or all four databases were regarded as eligible. Next, target long noncoding RNAs (lncRNAs) of the above-obtained miRNAs were predicted by StarBase and the LncBase module of the DIANA tool (http://carolina.imis.athena-innovation.gr/) (Karagkouni et al., 2020). Only the lncRNAs that were shared between the two databases were regarded as eligible. Finally, we selected lncRNA–mRNA interactions and miRNA–mRNA interactions that shared the same miRNAs to construct the ceRNA network and visualized it using Cytoscape software (Shannon et al., 2003) (Version 3.8.0, http://cytoscape.org).

### 2.5 Hub gene analysis

The cytoHubba plugin was applied to screen out the top ten genes of the above three ceRNA regulatory networks through seven different algorithms: MCC, Degree, Edge Percolated Component (EPC), EcCentricity, Closeness, Radiality, and Betweenness (Chin et al., 2014). UpSet R package was used to extract the overlapping genes obtained by the above seven different algorithms and visualize them (Conway et al., 2017). These overlapping genes were confirmed as the hub nodes.

## 3 Results

### 3.1 Identification of differentially expressed PCD-related genes

**FIGURE 1:**
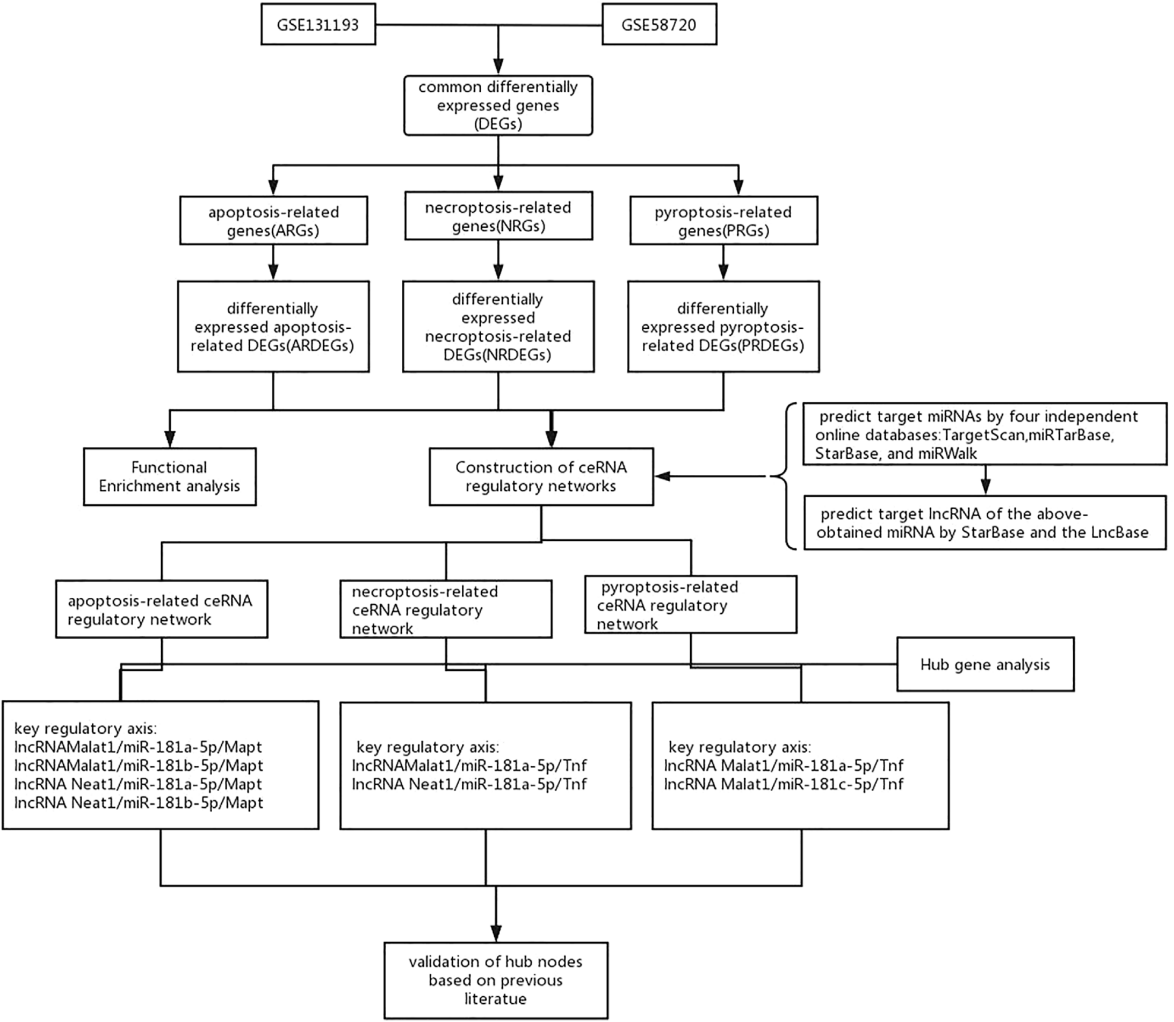
Flowchart of the analytical steps of this study.

**FIGURE 2:**
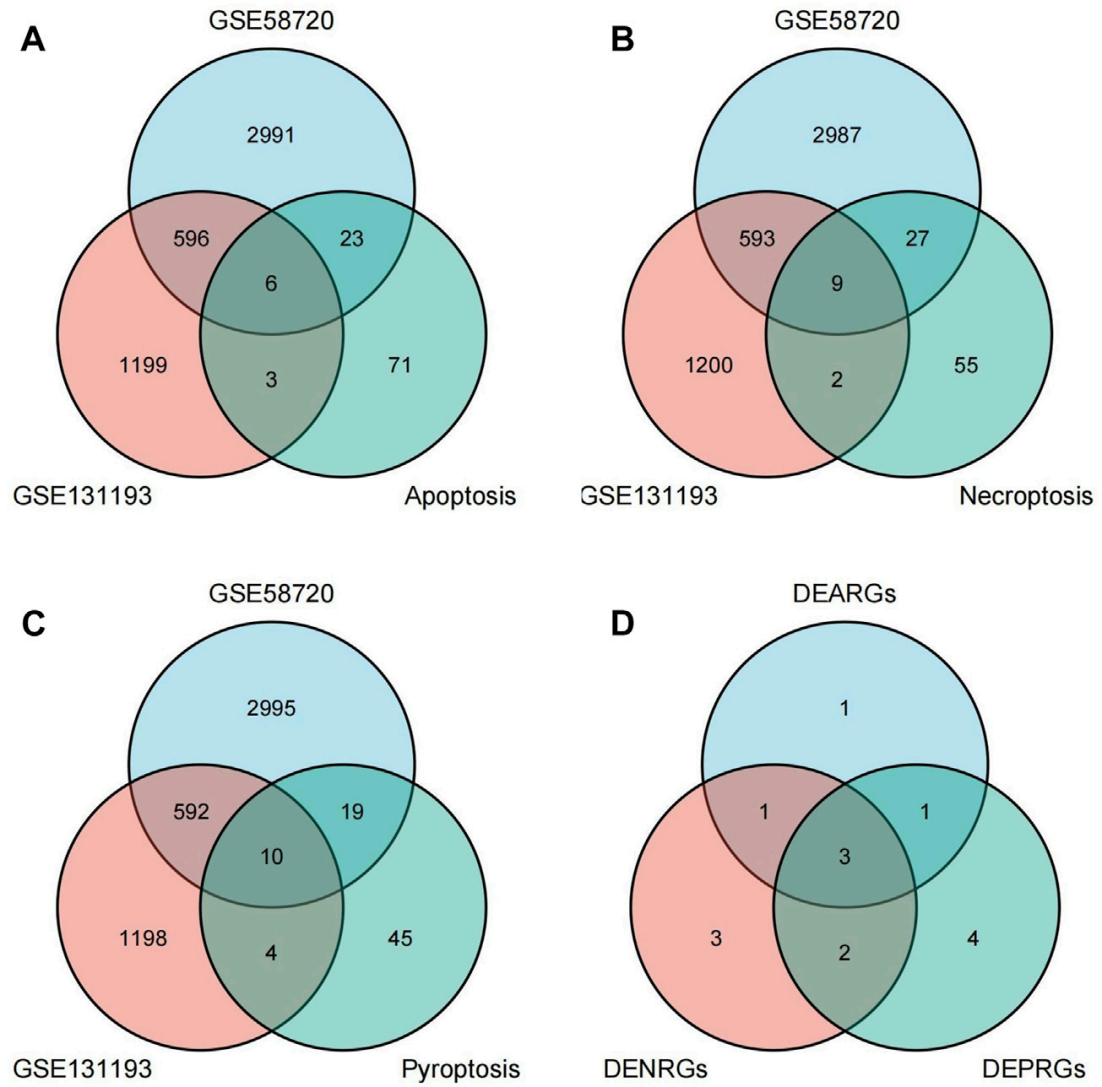
Identification of differentially expressed programmed cell death (PCD)-related genes. **(A)** Differentially expressed apoptosis-related genes (DEARGs) were identified by determining the overlap of datasets GSE58720 and GSE131193 with apoptosis-related genes. **(B)** Differentially expressed necroptosis-related genes (DENRGs) were identified by determining the overlap of datasets GSE58720 and GSE131193 with necroptosis-related genes. **(C)** Differentially expressed pyroptosis-related genes (DEPRGs) were identified by determining the overlap of datasets GSE58720 and GSE131193 with pyroptosis-related genes. **(D)** The overlapping genes were identified among DEARGs, DENRGs, and DEPRGs.

A flow chart of this study is shown in Figure 1. We first analyzed the two datasets GSE58720 and GSE131193 to identify the common DEGs in MCAO/R-operated mice at 24 h of reperfusion compared with matched controls. Then, we identified the common DEGs that overlapped with PCD (apoptosis, necroptosis, and pyroptosis)-related genes to obtain differentially expressed PCD-related genes (DEARGs, DENRGs, DEPRGs). A total of six DEARGs (Figure 2A), nine DENRGs (Figure 2B), and ten DEPRGs (Figure 2C) were identified. The six DEARGs included *Cd14*, *Zbp1*, *Tnfrsf10b*, *Bax*, *Mapt*, and *Pycard*, among which *Cd14, Zbp1, Tnfrsf10b, Bax*, and *Pycard* were upregulated in the dataset GSE58720 but downregulated in the dataset GSE131193, while *Mapt* showed the opposite pattern (Table 1). The nine DENRGs included *Cxcl1, Zbp1, Il1b, Tnf, Ripk3, Tnfrsf10b, Mlkl, Pycard,* and *Bax* (Table 1) and the ten DEPRGs included *Cd14, Zbp1, Il1b, Mefv, Tnf, Il1rn, Anxa2, Ccr5, Pycard*, and *Bax* (Table 1). All of these DENRGs and DEPRGs were upregulated in the dataset GSE58720 but downregulated in the dataset GSE131193. We also attempted to identify the common genes among the above three kinds of differentially expressed PCD-related genes and found that three genes, namely, *Zbp1, Bax*, and *Pycard*, overlapped among the three sets of differentially expressed PCD-related genes. *Cd14* was in common between DEARGs and DEPRGs, *Tnfrsf10b* was in common between DEARGs and DENRGs, while *Il1b* and *Tnf* were in common between DENRGs and DEPRGs (Figure 2D and Table 1).

**TABLE 1:** Differentially expressed PCD-related genes.

Gene symbol	Gene name	Expression in GSE58720	Expression in GSE131193	Belong to which kind of PCD related genes (DEARGs, DENRGs, DEPRGs)
Bax	BCL2-associated X protein	upregulated	downregulated	DEARG, DENRG, DEPRG
Pycard	PYD and CARD domain containing	upregulated	downregulated	DEARG, DENRG, DEPRG
Zbp1	Z-DNA binding protein 1	upregulated	downregulated	DEARG, DENRG, DEPRG
Tnfrsf10b	tumor necrosis factor receptor superfamily, member 10b	upregulated	downregulated	DEARG, DENRG
Il1b	interleukin 1 beta	upregulated	downregulated	DENRG, DEPRG
Tnf	tumor necrosis factor	upregulated	downregulated	DENRG, DEPRG
Cd14	CD14 antigen	upregulated	downregulated	DEARG, DEPRG
Mapt	microtubule-associated protein tau	downregulated	upregulated	DEARG
Cxcl1	chemokine (C-X-C motif) ligand 1	upregulated	downregulated	DENRG
Ripk3	receptor-interacting serine-threonine kinase 3	upregulated	downregulated	DENRG
Mlkl	mixed lineage kinase domain-like	upregulated	downregulated	DENRG
Mefv	Mediterranean fever	upregulated	downregulated	DEPRG
Il1rn	interleukin 1 receptor antagonist	upregulated	downregulated	DEPRG
Anxa2	annexin A2	upregulated	downregulated	DEPRG
Ccr5	chemokine (C-C motif) receptor 5	upregulated	downregulated	DEPRG

Note: PCD, programmed cell death; DEARG, differentially expressed apoptosis-related gene. DENRG, differentially expressed necroptosis-related gene; DEPRG, differentially expressed pyroptosis-related gene.

### 3.2 Functional enrichment analysis

**FIGURE 3:**
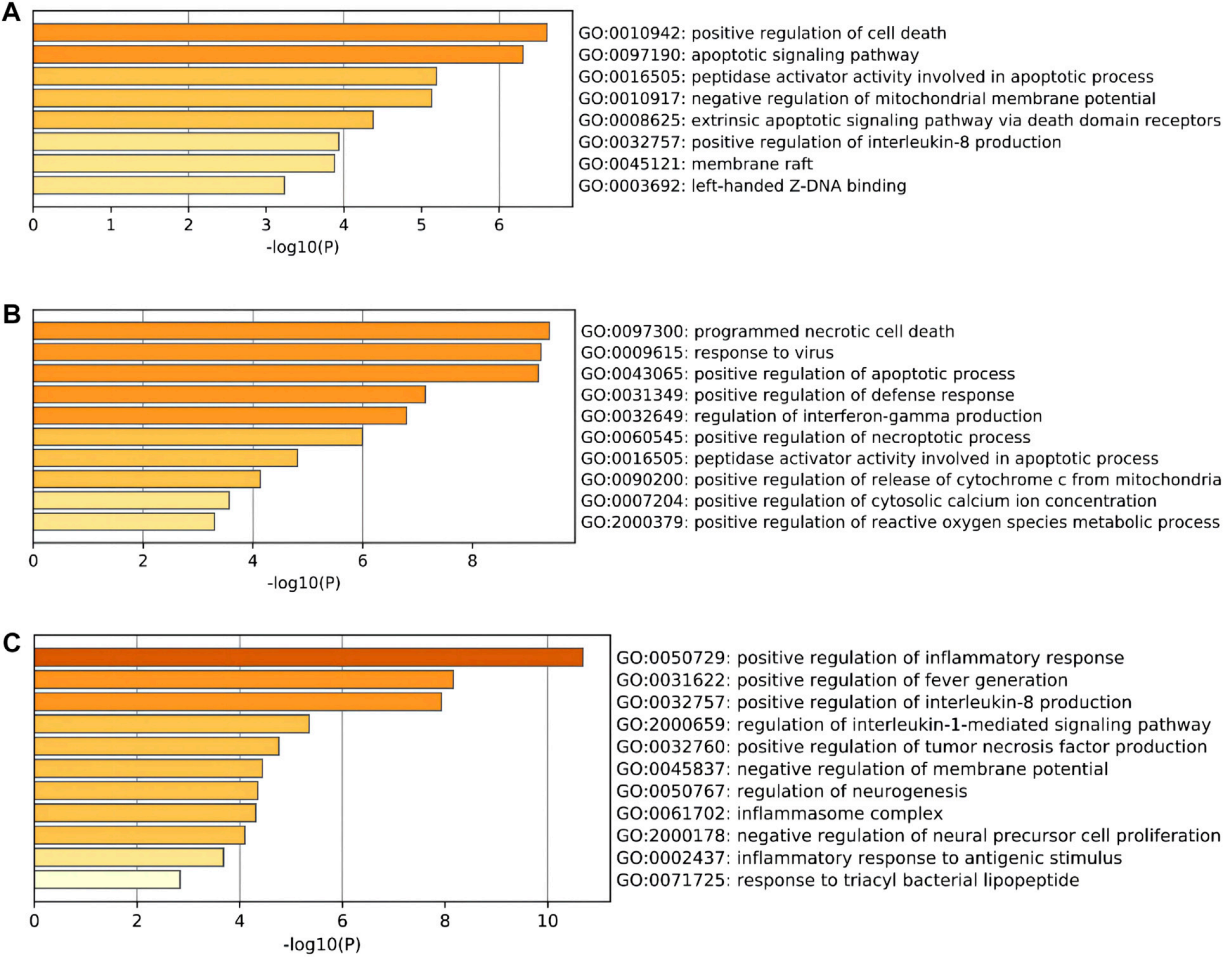
Gene Ontology (GO) enrichment analysis of these differentially expressed programmed cell death (PCD)-related genes. **(A)** Significantly enriched GO terms of differentially expressed apoptosis-related genes (DEARGs). **(B)** Significantly enriched GO terms of differentially expressed necroptosis-related genes (DENRGs). **(C)** Significantly enriched GO terms of differentially expressed pyroptosis-related genes (DEPRGs).

To further explore the potential functions of DEARGs, DENRGs, and DEPRGs, functional enrichment analysis was performed using the online database Metascape. The results of GO analysis revealed that the DEARGs were particularly associated with the positive regulation of cell death, apoptotic signaling pathway, negative regulation of mitochondrial membrane potential, extrinsic apoptotic signaling pathway via death domain receptors, positive regulation of interleukin-8 production, membrane rafts, and left-handed Z-DNA binding (Figure 3A and Table 2). The DENRGs were mainly associated with programmed necrotic cell death, response to virus, positive regulation of apoptotic process, necroptotic process, defense response, release of cytochrome c from mitochondria, cytosolic calcium ion concentration, reactive oxygen species metabolic process, and regulation of interferon-gamma production (Figure 3B and Table 3). The DEPRGs were particularly involved in the positive regulation of inflammatory response, tumor necrosis factor production, fever generation, interleukin-8 production, regulation of interleukin-1-mediated signaling pathway, regulation of neurogenesis, negative regulation of membrane potential and neural precursor cell proliferation, and inflammatory response to antigenic stimulus (Figure 3C and Table 4).

**TABLE 2:** Functional enrichment analysis of differentially expressed apoptosis-related genes (DEARGs).

Signifcant enriched GO terms of DEARGs				
Category	Term	Description	*p* value	Gene symbols
GO Biological Processes	GO:0010942	positive regulation of cell death	0.0000	Bax,Mapt, Tnfrsf10b,Zbp1,Pycard
GO Biological Processes	GO:0097190	apoptotic signaling pathway	0.0000	Bax,Mapt, Tnfrsf10b,Pycard
GO Biological Processes	GO:0010917	negative regulation of mitochondrial membrane potential	0.0000	Bax,Mapt
GO Biological Processes	GO:0008625	extrinsic apoptotic signaling pathway via death domain receptors	0.0000	Bax,Tnfrsf10b
GO Biological Processes	GO:0032757	positive regulation of interleukin-8 production	0.0001	Cd14,Pycard,Bax
GO Cellular Components	GO:0045121	membrane raft	0.0001	Cd14,Mapt, Tnfrsf10b
GO Molecular Functions	GO:0003692	left-handed Z-DNA binding	0.0006	Zbp1,Bax
Signifcant enriched pathways of DEARGs				
Reactome Gene Sets	R-MMU-109581	Apoptosis	0.0000	Bax,Cd14,Mapt, Tnfrsf10b
KEGG Pathway	mmu04217	Necroptosis	0.0000	Bax,Tnfrsf10b,Zbp1,Pycard
KEGG Pathway	mmu05417	Lipid and atherosclerosis	0.0000	Bax,Cd14,Tnfrsf10b,Pycard
Reactome Gene Sets	R-MMU-140534	Caspase activation via Death Receptors in the presence of ligand	0.0000	Cd14,Tnfrsf10b
KEGG Pathway	mmu05164	Influenza A	0.0000	Bax,Tnfrsf10b,Pycard
KEGG Pathway	mmu05134	Legionellosis	0.0001	Cd14,Pycard,Zbp1
Reactome Gene Sets	R-MMU-114294	Activation, translocation and oligomerization of BAX	0.0006	Bax,Cd14,Mapt
WikiPathways	WP493	Mapk signaling pathway	0.0009	Cd14,Mapt

**TABLE 3:** Functional enrichment analysis of differentially expressed necroptosis-related genes (DENRGs).

Signifcant enriched GO terms of DENRGs				
Category	Term	Description	*p* value	Symbols
GO Biological Processes	GO:0097300	programmed necrotic cell death	0.0000	Bax,Tnf,Ripk3,Mlkl,Il1b,Tnfrsf10b,Pycard, Cxcl1
GO Biological Processes	GO:0009615	response to virus	0.0000	Bax,Tnf,Ripk3,Zbp1,Pycard, Mlkl
GO Biological Processes	GO:0043065	positive regulation of apoptotic process	0.0000	Bax,Il1b,Tnf,Tnfrsf10b,Ripk3,Zbp1,Pycard
GO Biological Processes	GO:0031349	positive regulation of defense response	0.0000	Cxcl1,Il1b,Tnf,Zbp1,Pycard, Ripk3,Bax
GO Biological Processes	GO:0032649	regulation of interferon-gamma production	0.0000	Il1b,Tnf,Ripk3,Pycard,Zbp1,Bax,Cxcl1,Mlkl
GO Biological Processes	GO:0060545	positive regulation of necroptotic process	0.0000	Ripk3,Zbp1,Bax
GO Biological Processes	GO:0090200	positive regulation of release of cytochrome c from mitochondria	0.0001	Bax,Pycard, Ripk3
GO Biological Processes	GO:0007204	positive regulation of cytosolic calcium ion concentration	0.0003	Bax,Cxcl1,Il1b
GO Biological Processes	GO:2000379	positive regulation of reactive oxygen species metabolic process	0.0005	Cxcl1,Ripk3
Signifcant enriched pathways of DENRGs				
KEGG Pathway	mmu04217	Necroptosis	0.0000	Bax,Il1b,Tnf,Tnfrsf10b,Ripk3,Zbp1,Pycard, Mlkl
KEGG Pathway	mmu05417	Lipid and atherosclerosis	0.0000	Bax,Cxcl1,Il1b,Tnf,Tnfrsf10b,Pycard
KEGG Pathway	mmu04668	TNF signaling pathway	0.0000	Cxcl1,Il1b,Tnf,Ripk3,Mlkl
KEGG Pathway	mmu05134	Legionellosis	0.0000	Cxcl1,Il1b,Tnf,Pycard, Ripk3,Tnfrsf10b
KEGG Pathway	mmu04623	Cytosolic DNA-sensing pathway	0.0000	Il1b,Ripk3,Zbp1,Pycard
Reactome Gene Sets	R-MMU-5213460	RIPK1-mediated regulated necrosis	0.0000	Tnfrsf10b,Ripk3,Mlkl,Tnf
Reactome Gene Sets	R-MMU-75158	TRAIL signaling	0.0022	Tnfrsf10b
KEGG Pathway	mmu05167	Kaposi sarcoma-associated herpesvirus infection	0.0040	Bax,Cxcl1
Reactome Gene Sets	R-MMU-844456	The NLRP3 inflammasome	0.0044	Pycard, Cxcl1

**TABLE 4:** Functional enrichment analysis of differentially expressed pyroptosis-related genes (DEPRGs).

Signifcant enriched GO terms of DEPRGs				
Category	Term	Description	*p* value	Gene symbols
GO Biological Processes	GO:0050729	positive regulation of inflammatory response	0.0000	Ccr5,Il1b,Tnf,Mefv,Zbp1,Pycard,Cd14,Il1rn
GO Biological Processes	GO:0031622	positive regulation of fever generation	0.0000	Ccr5,Il1b,Tnf,Mefv, Pycard,Il1rn,Bax,Anxa2,Zbp1
GO Biological Processes	GO:0032757	positive regulation of interleukin-8 production	0.0000	Cd14,Il1b,Tnf,Pycard,Ccr5,Il1rn,Anxa2,Bax
GO Biological Processes	GO:2000659	regulation of interleukin-1-mediated signaling pathway	0.0000	Il1rn,Zbp1,Bax,Tnf,Pycard,Il1b
GO Biological Processes	GO:0032760	positive regulation of tumor necrosis factor production	0.0000	Cd14,Ccr5,Pycard,Bax,Il1b,Anxa2
GO Biological Processes	GO:0045837	negative regulation of membrane potential	0.0000	Bax,Il1rn,Anxa2
GO Biological Processes	GO:0050767	regulation of neurogenesis	0.0000	Anxa2,Ccr5,Il1b,Tnf,Cd14,Mefv
GO Cellular Components	GO:0061702	inflammasome complex	0.0000	Mefv, Pycard
GO Biological Processes	GO:2000178	negative regulation of neural precursor cell proliferation	0.0001	Ccr5,Il1b,Bax,Tnf,Mefv
GO Biological Processes	GO:0002437	inflammatory response to antigenic stimulus	0.0002	Il1rn,Tnf,Ccr5,Il1b,Anxa2
Signifcant enriched pathways of DEPRGs				
KEGG Pathway	mmu05132	*Salmonella* infection	0.0000	Bax,Anxa2,Cd14,Il1b,Tnf,Pycard,Zbp1,Ccr5
KEGG Pathway	mmu05135	*Yersinia* infection	0.0000	Il1b,Tnf,Mefv, Pycard
KEGG Pathway	mmu05152	Tuberculosis	0.0000	Bax,Cd14,Il1b,Tnf,Ccr5
Reactome Gene Sets	R-MMU-5660668	CLEC7A/inflammasome pathway	0.0000	Il1b,Pycard,Zbp1,Ccr5,Il1rn,Tnf
KEGG Pathway	mmu04061	Viral protein interaction with cytokine and cytokine receptor	0.0009	Ccr5,Tnf
Reactome Gene Sets	R-MMU-166020	Transfer of LPS from LBP carrier to CD14	0.0010	Cd14
Reactome Gene Sets	R-MMU-6798695	Neutrophil degranulation	0.0015	Anxa2,Cd14,Pycard

**FIGURE 4:**
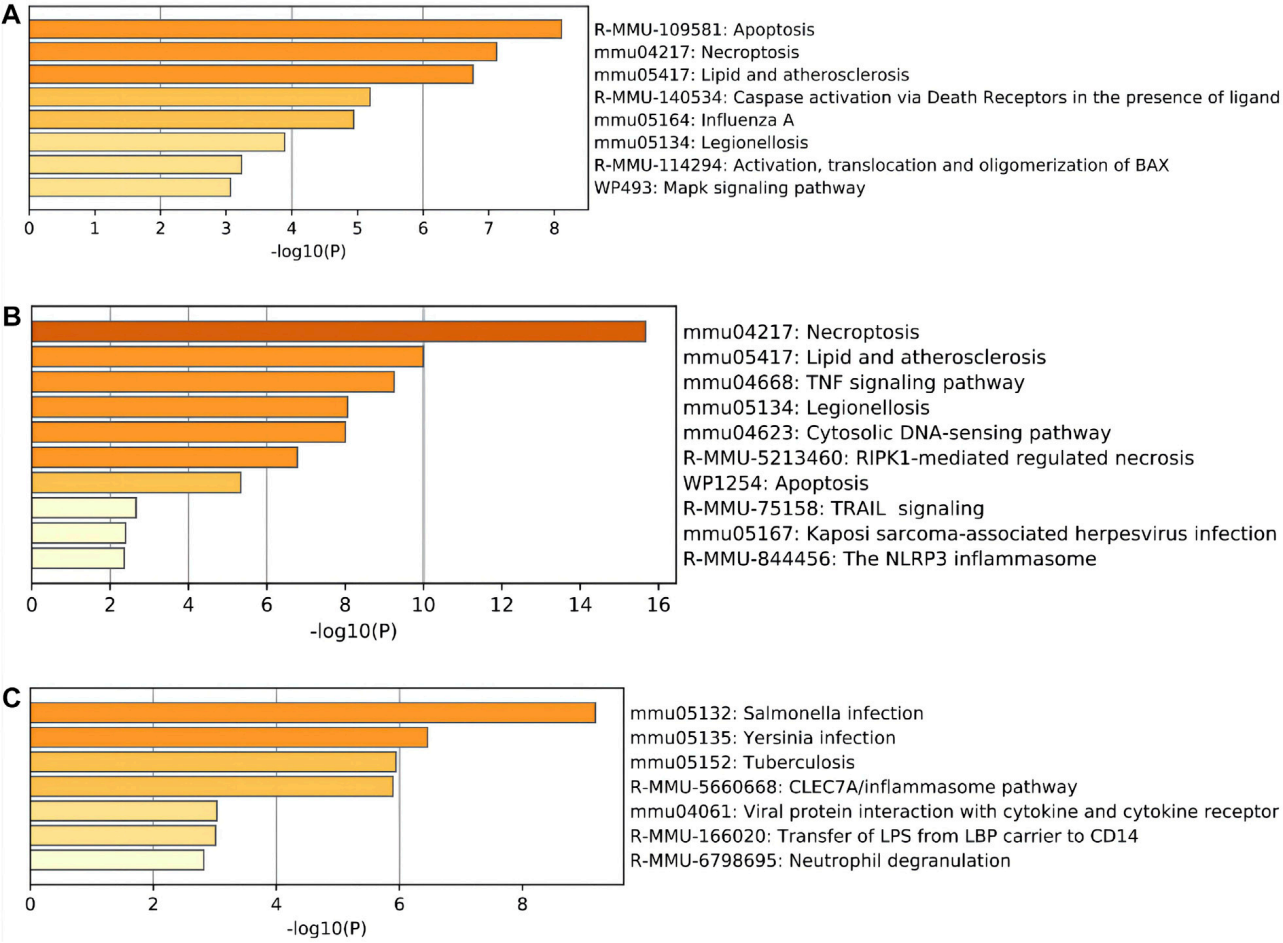
Pathway enrichment analysis of these differentially expressed programmed cell death (PCD)-related genes. **(A)** Significantly enriched pathways of differentially expressed apoptosis-related genes (DEARGs). **(B)** Significantly enriched pathways of differentially expressed necroptosis-related genes (DENRGs). **(C)** Significantly enriched pathways of differentially expressed pyroptosis-related genes (DEPRGs).

Moreover, regarding the results of pathway analysis, these revealed that the DEARGs were mainly associated with apoptosis, necroptosis, lipids and atherosclerosis, caspase activation via death receptors in the presence of ligand influenza A, legionellosis, activation, translocation and oligomerization of BAX, and the Mapk signaling pathway (Figure 4A and Table 2). The DENRGs were particularly involved in necroptosis, lipids and atherosclerosis, TNF signaling pathway, legionellosis, cytosolic DNA-sensing pathway, RIPK1-mediated regulated necrosis, TRAIL signaling, Kaposi sarcoma-associated herpesvirus infection, and the NLRP3 inflammasome (Figure 4B and Table 3). The DEPRGs were particularly associated with *Salmonella* infection, *Yersinia* infection, *tuberculosis*, CLEC7A/inflammasome pathway, viral protein interaction with cytokine and cytokine receptor, transfer of LPS from LBP carrier to CD14, and neutrophil degranulation (Figure 4C and Table 4).

### 3.3 Construction of PCD-related ceRNA networks

**FIGURE 5:**
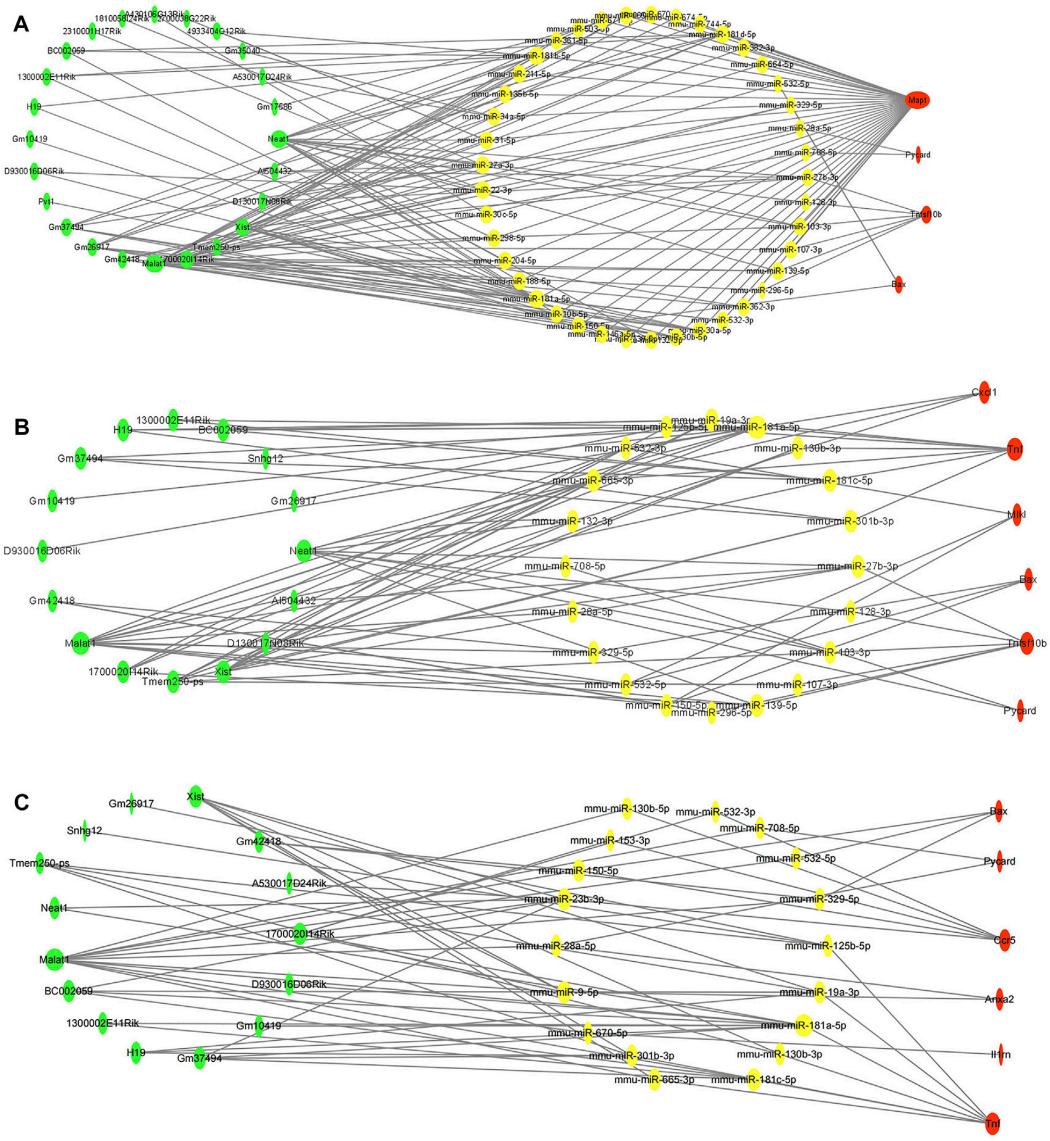
Construction of programmed cell death (PCD)-related ceRNA regulatory networks. **(A)** Apoptosis-related ceRNA regulatory networks. **(B)**. Necroptosis-related ceRNA regulatory networks. **(C)** Pyroptosis-related ceRNA regulatory networks. Red color denotes differentially expressed programmed cell death (PCD)-related genes, yellow color denotes eligible target miRNAs of these PCD-related genes, and green color denotes eligible target lncRNAs of miRNAs. The size of nodes represented closeness score calculated by cytoHubba plugin.

To clarify the potential molecular regulatory mechanisms of these differentially expressed PCD-related genes, we then constructed PCD-related ceRNA regulatory networks of lncRNA–miRNA–mRNA. First, four independent online databases, namely, TargetScan, miRTarBase, StarBase, and miRWalk, were used to predict the interactions between miRNAs and mRNAs. Only the miRNAs that were shared by any three or all four of the databases were regarded as eligible. A total of 133 miRNA–mRNA interactions for apoptosis-related ceRNA regulatory networks, 91 miRNA–mRNA interactions for necroptosis-related ceRNA regulatory networks, and 70 miRNA–mRNA interactions for pyroptosis-related ceRNA regulatory networks were obtained based on the above methods. Next, target lncRNAs of the above-obtained miRNAs were predicted by StarBase and the LncBase module of the DIANA tool; only the lncRNAs that were shared between the two databases were regarded as eligible. A total of 107 lncRNA–miRNA pairs for apoptosis-related ceRNA regulatory networks, 58 lncRNA–miRNA pairs for necroptosis-related ceRNA regulatory networks, and 49 lncRNA–miRNA pairs for pyroptosis-related ceRNA regulatory networks were identified. Then, the ceRNA networks were constructed using the miRNAs shared between the lncRNAs and mRNAs according to the ceRNA hypothesis. The apoptosis-related ceRNA regulatory network included 152 edges and 69 nodes (including 24 lncRNAs, 41 miRNAs, and 4 mRNAs) (Figure 5A). The necroptosis-related ceRNA regulatory network included 82 edges and 42 nodes (including 16 lncRNAs, 20 miRNAs, and 6 mRNAs) (Figure 5B). Finally, the pyroptosis-related ceRNA regulatory network included 69 edges and 39 nodes (including 15 lncRNAs, 18 miRNAs, and 6 mRNAs) (Figure 5C).

### 3.4 Hub gene analysis

**FIGURE 6:**
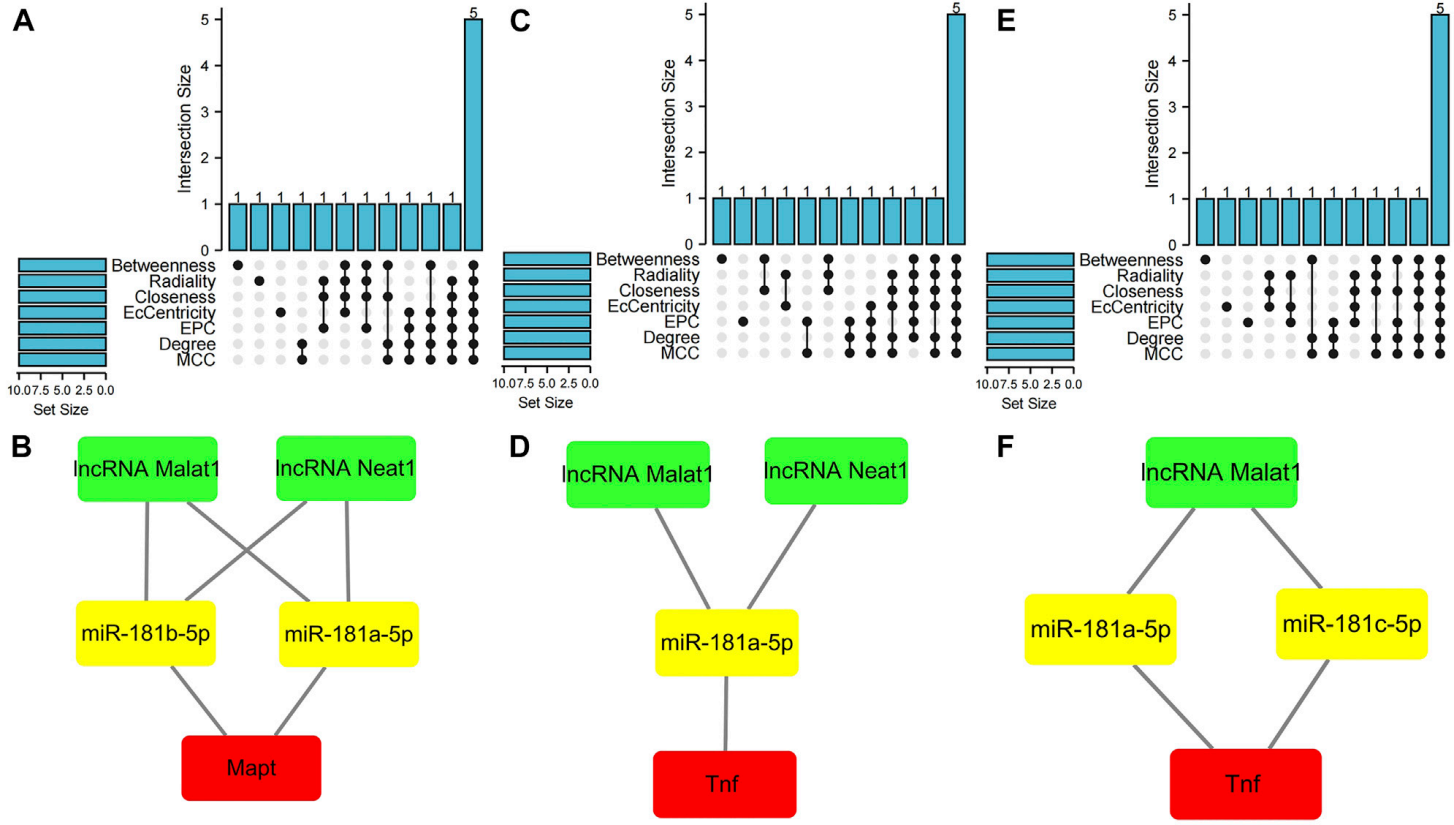
Hub gene analysis of three programmed cell death (PCD)-related ceRNA regulatory networks. **(A)** Hub nodes in the apoptosis-related ceRNA regulatory network that were shared by seven different algorithms. **(B)** Key ceRNA regulatory pathways for apoptosis-related ceRNA regulatory network. **(C)** Hub nodes in the necroptosis-related ceRNA regulatory network that were shared by seven different algorithms. **(D)** Key ceRNA regulatory pathways for necroptosis-related ceRNA regulatory network. **(E)** Hub nodes in the pyroptosis-related ceRNA regulatory network that were shared by seven different algorithms. **(F)** Key ceRNA regulatory pathways for pyroptosis-related ceRNA regulatory network. Red color denotes differentially expressed programmed cell death (PCD)-related genes, yellow color denotes miRNAs, and green color denotes lncRNAs.

The cytoHubba plugin was used to identify hub nodes of each of these PCD-related ceRNA regulatory networks based on the above methods. In the hub gene analysis, five hub nodes, namely, the mRNA Mapt, miR-181a-5p, miR-181b-5p, and the lncRNAs Malat1 and Neat1, were identified in the apoptosis-related ceRNA regulatory network (Figure 6A) and these hub nodes formed four ceRNA regulatory pathways, namely, lncRNA Malat1/miR-181a-5p/Mapt, lncRNA Malat1/miR-181b-5p/Mapt, lncRNA Neat1/miR-181a-5p/Mapt, and lncRNA Neat1/miR-181b-5p/Mapt (Figure 6B). Hub nodes in the necroptosis-related ceRNA regulatory network included mRNA Tnf, miR-181a-5p, lncRNA Malat1, lncRNA Xist, and lncRNA Neat1 (Figure 6C). These hub nodes formed two ceRNA regulatory pathways, namely, lncRNA Malat1/miR-181a-5p/Tnf and lncRNA Neat1/miR-181a-5p/Tnf (Figure 6D). Hub nodes in the pyroptosis-related ceRNA regulatory network included the mRNA Tnf, miR-181a-5p, miR-181c-5p, lncRNA Malat1, and lncRNA Xist (Figure 6E); these hub nodes formed two ceRNA regulatory pathways, namely, lncRNA Malat1/miR-181a-5p/Tnf and lncRNA Malat1/miR-181c-5p/Tnf (Figure 6F).

### 3.5 Validation of hub nodes in the programmed cell death-related ceRNA regulatory networks

To validate the hub nodes in these PCD-related ceRNA regulatory networks, we searched the literature and found that they were abnormally expressed in ischemic stroke. Shi et al. (Shi et al., 2021) found that the levels of acetylated tau (ac-MAPT) and phosphorylated tau (p-MAPT) increased in rats subjected to MCAO/R compared with that in the sham group. The protein and mRNA levels of total-tau (T-MAPT) showed no significant differences between the sham and MCAO/R groups. Basurto-Islas et al. (Basurto-Islas et al., 2018) observed higher phosphorylation of tau and total tau in MCAO/R mice. Other studies also reported that the hyperphosphorylation of tau increases during MCAO/R in animal models (Dong et al., 2014; Fujii et al., 2017). Tnf was also reported to be significantly upregulated in MCAO/R animal models and OGD/R cell models (Li et al., 2019; Zhang et al., 2021a; Zhou et al., 2021). Moreover, it was reported that miR-181a-5p was highly expressed in serum of ischemic stroke patients, brain tissues of MCAO/R mice, and an oxygen-glucose-deprivation/reoxygenation (OGD/R) N2a cell model (Ouyang et al., 2012; Wu et al., 2017; Song et al., 2021). Studies also reported that miR-181b-5p and miR-181c-5p expression was significantly decreased in cerebral ischemia *in vivo* and *in vitro* (Deng et al., 2016; Ma et al., 2016; Zhang et al., 2018; Meng et al., 2020). Accumulating evidence has also revealed that expression of the lncRNA Malat1 was upregulated after MCAO/R in rats and mice and OGD/R in different cells including primary neuronal cells, HT-22 cells, mouse astrocyte cells, and brain vascular endothelial cells (Xin and Jiang, 2017; Zhang et al., 2021b; Jia et al., 2021; Tan et al., 2021). Moreover, a recent study reported that the lncRNA Malat1 significantly increased in the blood of ischemic stroke patients compared with the level in normal controls (Tan et al., 2021). Furthermore, several studies reported that the lncRNA Neat1 was increased in an MCAO/R animal model, an OGD/R-induced cell model, and ischemic stroke patients (Ni et al., 2020; Zhang et al., 2021c; Jin et al., 2021). Another recent study reported that expression of the lncRNA Neat1 was significantly decreased in OGD/R-induced BV-2 and N2a cells compared with that in control cells (Zhou et al., 2022). The lncRNA XIST was also reported to be highly expressed in an MCAO/R-treated animal model and an OGD/R-treated cell model (Zhang et al., 2021d; Wang et al., 2021; Xiong et al., 2021). Wang et al. also reported that the lncRNA XIST was upregulated in brain tissues under MCAO/R treatment and in OGD/R-treated PC12 cells (Wang et al., 2021). Furthermore, Xiong et al. found that XIST was significantly highly expressed in SH-SY5Y cells after OGD/R treatment (Xiong et al., 2021). Finally, another study identified that XIST expression was upregulated in the brain tissues of an I/R mouse model and OGD/R-induced N2a cells (Zhang et al., 2021d). The findings of these previous studies are in accordance with our results, indicating the robustness of our analysis.

## 4 Discussion

Accumulating evidence supports the involvement of PCD pathways in the pathogenesis of ischemic stroke and highlights the importance of each form of cell death. However, the specific mechanisms underlying them remain incompletely clarified. There is also currently a lack of specific neuroprotective drugs in clinical practice. Nonetheless, increasing studies have indicated significant crosstalk among these PCD pathways. Therefore, we applied bioinformatic analysis to identify differentially expressed PCD-related genes during cerebral I/R injury and investigated their potential regulatory axes by constructing ceRNA networks. This may contribute to elucidating the molecular mechanisms behind these PCD pathways and provide a basis for developing novel therapeutic targets against cerebral I/R injury.

A total of six DEARGs, nine DENRGs, and ten DEPRGs were identified in this study. Among them, three genes, namely, Bax, Zbp1, and Pycard, overlapped among these three sets of genes, indicating that they may play key roles in the crosstalk among these PCD pathways. The protein encoded by the *Bax* gene belongs to the BCL2 protein family and is regarded as the fundamental effector of the intrinsic apoptotic pathway (Spitz and Gavathiotis, 2022). Numerous studies have indicated that Bax-dependent initiation and activation of subsequent apoptotic pathways play critical roles in ischemic brain injury (Li et al., 2021; Tu and Hu, 2021). In addition, it has been suggested that inhibition of Bax function may provide a new strategy for neuroprotection and functional improvement against cerebral ischemia (Han et al., 2011). Recently, some studies also reported that Bax is a key regulator of caspase-independent necroptosis and pyroptosis (Cabon et al., 2012; Hu et al., 2020). However, whether Bax is involved in necroptosis and pyroptosis in cerebral ischemia–reperfusion injury has remained unclear, so further research on this issue is needed. The gene *Pycard* encodes the adaptor protein ASC, which comprises two protein–protein interaction domains: an N-terminal PYRIN-PAAD-DAPIN domain (PYD) and a C-terminal caspase-recruitment domain (CARD) (Hoss et al., 2017). Previous studies demonstrated that ASC was upregulated in an ischemic stroke model and played a key role in cerebral ischemia–reperfusion injury by participating in the inflammatory response and cell death, including apoptosis, necroptosis, and pyroptosis (Meng et al., 2019; Liang et al., 2020; Xu et al., 2021a). The gene *Zbp1* encodes Z-DNA binding protein 1 with two Zα domains, which is a critical innate immune sensor of not only viral RNA products but also endogenous nucleic acid ligands (Zhang et al., 2020). Previous studies showed that Zbp1 plays a role in the innate immune response by binding to foreign DNA and inducing type I interferon production (Jiao et al., 2020). In addition, in response to influenza virus infection, it could induce cell death in the form of pyroptosis, apoptosis, and necroptosis, that is, PANoptosis (Zheng and Kanneganti, 2020). However, the role of Zbp1 in cerebral I/R injury remains unknown. Recent studies demonstrated that Zbp1 and ASC are components of the PANoptosome (Zheng and Kanneganti, 2020; Lee et al., 2021). Yan et al. (Yan et al., 2022) also indicated that PANoptosis is observed in ischemic brain injury. Based on previous related studies, we speculated that these three genes might be components of the PANoptosome and be involved in PANoptosis in cerebral I/R injury. Our study further confirmed previous findings supporting the hypothesis that these three genes might be involved in PANoptosis and crosstalk among apoptosis, necroptosis, and pyroptosis in cerebral I/R injury, thus providing new targets for neuroprotection.

Functional enrichment analyses were performed to obtain a more in-depth understanding of the differentially expressed PCD-related genes. The results showed that these genes were not only particularly associated with corresponding PCD-related biological processes and pathways, but also involved in other biological processes and pathways, such as inflammatory response and reactive oxygen species metabolic process. This indicates that these genes have different functions under particular circumstances and that there might be crosstalk among these biological processes. These results are in line with previous studies that revealed significant crosstalk between PCD and inflammatory response (Jayaraj et al., 2019).

In recent years, increasing studies have suggested that the regulatory network composed of lncRNAs, miRNAs, and mRNAs plays a critical role in the mechanisms underlying cerebral ischemia–reperfusion injury (Xu et al., 2021b). To better understand the molecular regulatory mechanisms of these differentially expressed PCD-related genes, we constructed PCD-related ceRNA regulatory networks and performed hub gene analysis to identify key nodes in these networks. The gene *Mapt* was found to be a hub node of the apoptosis-related ceRNA regulatory network. It can encode several isoforms of tau protein as a result of complex, regulated alternative splicing of its messenger RNA (Zhang et al., 2009). *Mapt* transcripts are differentially expressed in the nervous system, depending on the stage of neuronal maturation and neuron type. *Mapt* gene mutations have been shown to be associated with several neurodegenerative disorders (Michalicova et al., 2020). In recent years, increasing evidence has demonstrated that *Mapt* plays a role in ischemic stroke. In addition, Basurto-Islas et al. (Basurto-Islas et al., 2018) found that a large amount of hyperphosphorylated MAPT (Ser262/356) was colocalized with apoptotic cells in MCAO/R-treated mice. Moreover, Fujii et al. (Fujii et al., 2017) illustrated that the knockout of MAPT reduced infarct area and alleviated symptoms of neurological deficit. A recent study also showed that astragaloside IV exerted neuroprotective effects in rats with cerebral ischemia/reperfusion (CIR) injury, probably through the Sirt1/Mapt pathway (Shi et al., 2021). In this study, the gene *Tnf* was identified as a hub node in both the necroptosis-related ceRNA regulatory network and the pyroptosis-related ceRNA regulatory network. This gene encodes the multifunctional proinflammatory cytokine TNF-α, which belongs to the tumor necrosis factor (TNF) superfamily (Watters and O'Connor, 2011). It can bind to its surface receptors and functions through their activation. Generally, TNF is a classical activator of necroptosis that binds to its receptor to recruit RIPK1, which interacts with RIPK3 to form necrosome and phosphorylate MLKL to mediate necroptosis in the absence of caspases-8 (Green, 2019). Studies have shown that the level of TNF-α was elevated in ischemic stroke and it has been implicated in cerebral I/R injury, exerting effects by regulating the inflammatory response and PCD pathways including apoptosis, necroptosis, and pyroptosis (Hallenbeck, 2002; Maddahi et al., 2011), which is consistent with our results. Many studies have demonstrated that the inhibition of TNF signaling pathways may have neuroprotective effects against cerebral I/R injury. For example, Zhang et al. showed that preconditioning with Carbonisatus significantly decreased the levels of TNF-α and IL-6, reduced ischemic lesion volume, and improved neurological deficits in MCAO/R rats (Zhang et al., 2021a).

MicroRNAs (miRNAs) are conserved small regulatory noncoding RNAs of about 20–22 bp in length. They can regulate protein expression by binding to the 3′ untranslated region (3′UTR) of their target genes, degrading or inhibiting their expression (Krol et al., 2010). Numerous studies have shown that miRNAs are involved in the regulation of PCD pathways in many diseases, including ischemic stroke (Ghafouri-Fard et al., 2020). The hub gene analysis in this study demonstrated that mir-181a-5p was a hub node in all of the above-mentioned three PCD-related ceRNA networks, and that mir-181b-5p was a hub node of the apoptosis-related ceRNA regulatory network and mir-181c-5p was a hub node of the pyroptosis-related ceRNA regulatory network. miR-181a-5p, miR-181b-5p, and miR-181c-5p all belong to the miR-181 family and their aberrant expression has been associated with various diseases including stroke, neurodegeneration, and cancer (Indrieri et al., 2020). Previous studies suggested that the miR-181 family participates in the regulation of a range of biological processes including cell proliferation (Huo et al., 2016), apoptosis (Zhang et al., 2018), autophagy (Guo et al., 2019), and immune and inflammatory responses (Hutchison et al., 2013; Lu et al., 2019). Moreover, several studies have demonstrated that the inhibition of miR-181a-5p played a neuroprotective role in cerebral ischemic injury, as evidenced by reductions in cell apoptosis, pyroptosis, and cerebral infarction area (Moon et al., 2013; Stary et al., 2017; Yan et al., 2020; Song et al., 2021). However, the roles of miR-181b-5p and miR-181c-5p in cerebral ischemia have remained controversial. Peng et al. showed that downregulated miR-181b played a neuroprotective role against ischemic injury through negatively regulating HSPA5 and UCHL1 protein levels (Peng et al., 2013). In addition, Zhang et al. suggested that the downregulation of miRNA-181b protects against cerebral ischemic injury via the inhibition of NF-κB-mediated inflammatory and apoptotic responses (Zhang et al., 2018). In contrast, another two reports demonstrated the possible neuroprotective effects of increased miR-181b in ischemia-caused neuronal cell apoptosis and mechanical repair of brain tissue (Deng et al., 2016; Liu et al., 2016). Most studies supported the assertion that miR-181c-5p plays a positive role in brain injury caused by cerebral ischemia–reperfusion and that its overexpression can inhibit brain injury caused by ischemic stroke through regulating proliferation, inflammatory response, and apoptosis of neuronal cells (Zhang et al., 2019; Cao et al., 2020; Bu et al., 2021). However, in two other studies, the opposite conclusions were drawn. Specifically, Ma et al. (Ma et al., 2016) found a positive correlation between the NIHSS score and miR-181c level, and showed that plasma miR-181c concentration was positively correlated with the number of neutrophils and blood platelet count and negatively correlated with the number of lymphocytes. They also found that miR-181c promoted the apoptosis of BV2 and Neuro-2a cells and aggravated brain ischemia–reperfusion injury in a mouse model of stroke via the modulation of pro- and anti-apoptotic proteins. Moreover, a recent study showed that downregulated miR-181c ameliorated cerebral ischemic injury via increasing the expression of c-Fos and its downstream genes (Meng et al., 2020). Taken together, these findings indicated that mir-181a-5p, miR-181b-5p, and miR-181c-5p are all involved in the mechanism of cerebral I/R injury, but might play different roles depending on the specific target gene to which they bind.

lncRNAs are the most abundant noncoding RNAs (ncRNAs). They are greater than 200 bp in length, lack protein-coding function, and are associated with a variety of neurological diseases including ischemic stroke (Wu et al., 2013; Bao et al., 2018). Our hub analysis identified the lncRNA Malat1 as a hub node in all three PCD-related ceRNA regulatory networks, which is consistent with previous studies, indicating its critical role in regulating PCD pathways. Malat1 is known as a long intergenic noncoding RNA and is highly abundant in the nervous system. Accumulating evidence has indicated that this lncRNA plays vital roles in regulating various physiological processes, including apoptosis, autophagy, immune and inflammatory responses, and endothelial dysfunction of ischemic stroke (Wang et al., 2022). The expression of Malat1 was also found to be upregulated in ischemic stroke, while its downregulation was shown to improve the neurological deficit score and reduce neuronal apoptosis and the size of cerebral infarction by regulating miR-211-5p to in turn regulate the expression of COX-2 (Tan et al., 2021). Other studies also demonstrated that the inhibition of Malat1 expression could protect against cerebral I/R injury by alleviating neuronal apoptosis, endoplasmic reticulum stress, and inflammation (Shi et al., 2019; Cao et al., 2020; Jia et al., 2021). Moreover, it was reported that Malat1 was highly expressed in OGD/R-induced astrocyte injury models, and that its silencing protected against cerebral ischemia–reperfusion injury by downregulating AQP4 levels via miR-145 (Wang et al., 2020). In contrast, some studies supported the neuroprotective role of Malat1 in cerebral ischemia–reperfusion injury. For example, Xin et al. (Xin and Jiang, 2017) found that Malat1 could protect human brain vascular endothelial cells from OGD/R-induced apoptosis via a PI3K-dependent mechanism. Another study showed that mice with lncRNA Malat1 KO presented larger brain infarct size and worse neurological scores, indicating that Malat1 plays critical protective roles in ischemic stroke via anti-apoptotic and anti-inflammatory effects in the brain microvasculature (Zhang et al., 2017). Accumulating evidence has also indicated that Malat1 is an important regulator of pyroptosis in various diseases (Song et al., 2019; Shu et al., 2021). However, the specific roles and mechanisms by which Malat1 regulates pyroptosis and necroptosis in cerebral ischemia–reperfusion injury have remained unclear. In addition, the lncRNAMalat1/miR-181a-5p/Mapt regulatory axis, lncRNAMalat1/miR-181b-5p/Mapt regulatory axis, and lncRNA Malat1/miR-181a-5p/Tnf regulatory axis were not previously reported to be involved in cerebral I/R injury, so they need further investigation. In this study, the lncRNA Neat1 was also identified as a hub node in both apoptosis-related and necroptosis-related ceRNA regulatory networks. Recently, increasing evidence has shown that this lncRNA plays an essential role in physiological and pathological responses in ischemic stroke (Ni et al., 2020; Jin et al., 2021). Li et al. (Li et al., 2020) found that the expression of Neat1 was elevated in patients with ischemic stroke compared with that in controls, and that lncRNA Neat1 expression positively correlated with NIHSS score and inflammatory factors and could predict an increased risk of recurrence/death. Ni et al. (Ni et al., 2020) also showed that Neat1 knockdown alleviated OGD/R-induced apoptosis and increased neuronal viability. Another study demonstrated that Gastrodin significantly alleviated cerebral I/R injury by regulating the lncRNA Neat1/miR-22-3p axis; it also showed that the overexpression of Neat1 promoted neuronal pyroptosis (Zhang et al., 2021c). Previous studies also reported that the downregulation of Neat1 could exert anti-inflammatory effects in cerebral I/R injury (Han and Zhou, 2019; Jin et al., 2021). Taken together, these findings indicate that the lncRNA Neat1 might play crucial roles in PCD pathways and inflammation in cerebral I/R injury and is a potential therapeutic target. Another lncRNA identified as a hub node in both necroptosis-related ceRNA and pyroptosis-related regulatory networks is Xist. Previous studies confirmed that Xist contributes to cerebral I/R injury through modulating cell apoptosis, ROS production, and inflammation. Wang et al. also demonstrated that the silencing of XIST protected against cerebral I/R injury by inhibiting neuronal deficit and inflammation via the miR-362/ROCK2 axis (Wang et al., 2021). In addition, Xiong et al. reported that XIST reduced cell viability and induced cell apoptosis via modulating miR-486-5p and GAB2, which promoted cerebral I/R injury (Xiong et al., 2021). Another two studies also indicated that knockdown of XIST inhibited brain injury by suppressing apoptosis and ROS production (Zhang et al., 2021d; Weng et al., 2021). Moreover, a recent study illustrated that Xist was involved in the regulation of pyroptosis in MCAO/R-treated rats and OGD/R-treated rat brain microvascular endothelial cells (Guo et al., 2022). Nevertheless, the molecular roles and regulatory mechanisms of XIST in necroptosis and pyroptosis in cerebral I/R injury have not been fully elucidated and require further research.

In summary, we analyzed datasets GSE131193 and GSE58720 to identify PCD-related genes signature and potential regulatory axes in cerebral I/R injury and the results were validated through previous work. To our knowledge, this was the first study to focused on PCD (apoptosis, necroptosis, and pyroptosis)-related genes and potential regulatory axes in cerebral I/R injury, which might have profound significance for ischemia stroke. We identified hub nodes and seven key ceRNA regulatory axes that has never been reported before in ischemia stroke, which may contribute to elucidating the molecular mechanisms and provide a basis for developing novel therapeutic targets against cerebral I/R injury. Further *in vivo* and *in vitro* studies should be conducted to verify these regulatory axes.

There were several limitations to this study that should be acknowledged. First, the PCD (apoptosis, necroptosis, and pyroptosis)-related genes included in this study were mainly identified in previous studies, so some unreported related genes may have been ignored or excluded. Second, both lncRNAs and miRNAs were obtained by online database prediction because neither lncRNA nor miRNA datasets on adult mouse brain tissues after transient focal ischemia at 24 h of reperfusion and matched control samples were available. In future studies, if available, lncRNA and miRNA datasets should be analyzed simultaneously to increase the reliability of the results. Third, the selected datasets were performed in different laboratories and therefore, the differences in sample preparation, sample collection methods, and microarray platforms might influence the results. Finally, our hypothesized potential binding affinity among lncRNAs, miRNAs, and mRNAs should be subjected to further experimental investigation.

## 5 Conclusion

Taken together, our findings indicated that these PCD pathways (apoptosis, necroptosis, pyroptosis, and PANoptosis) and crosstalk among them might be involved in ischemic stroke. And the key nodes and regulatory axes identified in this study might play vital roles in regulating the above processes, which may offer new insights into the potential mechanisms underlying cell death during cerebral I/R injury and provide new therapeutic targets for neuroprotection.

## Data Availability

The original contributions presented in the study are included in the article/Supplementary Material, further inquiries can be directed to the corresponding authors.
